# Beyond the bulk: overview and novel insights into the dynamics of muscle satellite cells during muscle regeneration

**DOI:** 10.1186/s41232-024-00354-1

**Published:** 2024-09-26

**Authors:** Woo Seok Byun, Jinu Lee, Jea-Hyun Baek

**Affiliations:** School of Life Science, Handong Global University, Pohang, Gyeongbuk 37554 Republic of Korea

**Keywords:** Single-cell technology, Skeletal muscle satellite cell, Tissue regeneration, Macrophage, Cellular dynamics

## Abstract

Skeletal muscle possesses remarkable regenerative capabilities, fully recovering within a month following severe acute damage. Central to this process are muscle satellite cells (MuSCs), a resident population of somatic stem cells capable of self-renewal and differentiation. Despite the highly predictable course of muscle regeneration, evaluating this process has been challenging due to the heterogeneous nature of myogenic precursors and the limited insight provided by traditional markers with overlapping expression patterns. Notably, recent advancements in single-cell technologies, such as single-cell (scRNA-seq) and single-nucleus RNA sequencing (snRNA-seq), have revolutionized muscle research. These approaches allow for comprehensive profiling of individual cells, unveiling dynamic heterogeneity among myogenic precursors and their contributions to regeneration. Through single-cell transcriptome analyses, researchers gain valuable insights into cellular diversity and functional dynamics of MuSCs post-injury. This review aims to consolidate classical and new insights into the heterogeneity of myogenic precursors, including the latest discoveries from novel single-cell technologies.

**Supplementary Information:**

The online version contains supplementary material available at 10.1186/s41232-024-00354-1.

## Introduction

Skeletal muscle exhibits remarkable regenerative potential, with complete restoration of structure and function occurring in less than a month following severe acute damage in humans and mice [1, 2]. Notably, skeletal muscle regeneration depends on the timely coordinated interaction of heterogeneous cell types, signaling molecules, structural cues, and physicochemical properties to be successful. Following muscle injury, the plasma membrane surrounding myofibers degenerates, releasing cellular contents and chemotactic factors into the extracellular space. These prompt mast cells, neutrophils, and inflammatory monocytes to infiltrate the injury site even before 3 h post-injury [3]. Infiltrating monocytes, in turn, triggers a cascade of cellular responses to modulate the activation and proliferation of muscle stem cells, also known as muscle satellite cells (MuSCs) [4, 5]. During the first 24 h post-injury, MuSCs differentiate into myoblasts, which either divide a limited number of times to maintain the stem cell pool or terminally differentiate into myocytes, ultimately fusing to form multinucleated myotubes [6, 7]. At this stage, the extracellular matrix (ECM) is constantly remodeled in response to the secretion of its constituents, fibrogenic cytokines, and growth factors from interstitial fibroblasts and fibro-adipogenic progenitors (FAPs) [8, 9]. The connective tissue re-establishes the firm attachment of myofiber ends and rebuilds the myotendinous junction (MTJ) where myofibers attach to tendons [10]. Finally, the muscle repair is completed when the blood supply to the injury site is restored through revascularization and myofibers become innervated via neuromuscular junction (NMJ) formation [11].

**Fig. 1:**
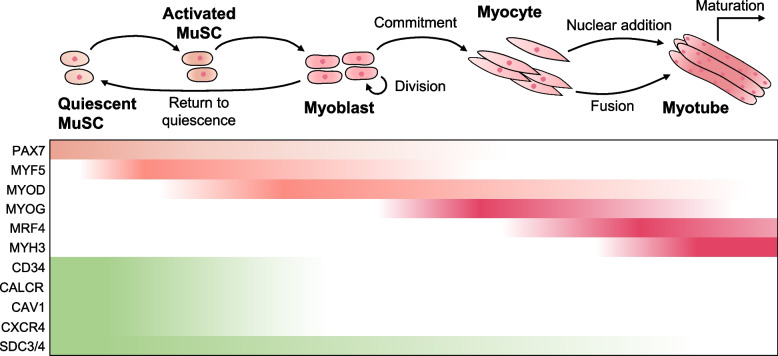
Cell state and division of MuSCs. The developmental stages of MuSCs can be delineated by the hierarchical protein expression of the paired box, myogenic regulatory factors (MRFs), MYH3, and various surface markers

Historically, researchers have relied on the expression patterns of surface markers such as CD34, calcitonin receptor (CALCR), caveolin 1 (CAV1), C–X–C chemokine receptor type 4 (CXCR4), and syndecan-3/4 (SDC3/4) to identify and isolate MuSCs (Fig. 1) [12–15]. However, increasing evidence suggests that MuSCs exist in different phenotypes, which has led to the recognition of the functional and transcriptional heterogeneity among MuSCs, thus impeding the assessment of their dynamics during muscle regeneration using individual markers [16, 17]. Nowadays, numerous novel single-cell analysis methods are available, bringing significant advancements to muscle research. While bulk analyses provide an aggregated view of cell populations, these novel state-of-the-art methods provide comprehensive profiling of individual cells, enabling us to dissect the complex heterogeneity of myogenic precursors and their contributions to skeletal muscle growth and repair. This feature not only enhances our ability to identify key cellular interactions and molecular pathways of muscle regeneration but also broadens our fundamental understanding, paving the way for new approaches to treating muscle-related conditions.

Recent publications on single-cell transcriptome analysis of injured skeletal muscle have been particularly illuminating, revealing previously unrecognized diversity among myogenic precursors and their progeny (Table 1). Accordingly, this review aims to consolidate the latest findings and discussions in muscle stem cell research, focusing on the biological insights unveiled by single-cell technologies. In this review, we wish to highlight the technical challenges and caveats of single-cell technology while contemplating their promising future and potential impact on the field.

**Table 1:** List of single-cell studies on MuSC using sorted cells or whole muscles

Studies	Omics type	Cell Isolation procedure	Injury site	Injury model (dpi)	Ref
Dell’Orso et al., 2019	scRNA-seq	FACS (MuSC)	HL	NTX (0, 2.5)	[18]
De Micheli et al., 2020	scRNA-seq	Whole muscle + FACS (Live)	TA	NTX (0, 2, 5, 7)	[19]
	scRNA-seq	Whole muscle	TA	NTX (0, 2, 5, 7)	
Lee et al., 2022	scRNA-seq	Whole muscle	TA	BaCl_2_ (4)	[20]
Oprescu et al., 2020	scRNA-seq	Whole muscle + FACS (Live)	TA	CTX (0, 0.5, 2, 3.5, 5, 10, 21)	[21]
Yartseva et al., 2020	scRNA-seq	FACS (MuSC)	TA	CTX (0, 4)	[22]
Machado et al., 2021	scRNA-seq	FACS (MuSC)	TA	BaCl_2_ (0, 2 h, 4 h)	[23]
McKellar et al., 2021	scRNA-seq	Whole muscle	TA	NTX (0, 1, 2, 3.5, 5, 7)	[24]
	Spatial	-	TA	NTX (2, 5, 7)	
Xu et al., 2021	scRNA-seq	Whole muscle	TA	GLY (5)	[25]
Cutler et al., 2022	scRNA-seq	Whole muscle	TA	BaCl_2_ (0, 4, 7)	[26]
Okafor et al., 2023	scRNA-seq	Whole muscle + FACS (CD31^−^CD45^−^)	HL	BaCl_2_ (3)	[27]
Southerland et al., 2023	scRNA-seq	Whole muscle + FACS (Live)	HL	IR (0, 1, 3, 7)	[28]
Young et al., 2022	scRNA-seq	Whole muscle	TA	CTX (0, 5)	[29]

*Abbreviations*: *TA* Tibialis anterior, *HL* Hind limb, *CTX* Cardiotoxin, *GLY* Glycerol, *IR* Ischemia–reperfusion

## Muscle satellite cells

MuSCs represent a specialized type of adult stem cell localized within the skeletal muscle tissue. Under homeostatic conditions, MuSCs reside between the basal lamina of the basement membrane and the myofiber plasmalemma [30]. They are maintained in a reversible state called quiescence. The quiescent state is crucial for preserving stem cell properties, preventing uncontrolled activation and exhaustion of the stem cell pool while avoiding the accumulation of DNA damage and senescence [31]. Quiescent MuSCs commonly express paired box 7 (PAX7), a canonical myogenic marker that represses genes involved in muscle differentiation and is essential for orchestrating proper muscle regeneration [32]. Additionally, they exhibit a repertoire of cell surface markers, including SDC3/4, α7β1 integrin, CD34, c-Met, NCAM, VCAM1, N-cadherin, M-cadherin, CXCR4, CALCR, and CAV1. This molecular profile primes them for swift activation in response to muscle injury [12]. Of note, PAX7 is also expressed in activated MuSCs, albeit at a relatively lower level [33–35].

Surface markers specific for quiescent MuSCs have been integral in primary cell isolation by fluorescence-activated cell sorting (FACS) from the murine skeletal muscle. Studies from various laboratories have established that MuSCs can be isolated through the positive selection of CD34^+^α7-integrin^+^ [36, 37], CXCR4^+^β1-integrin^+^ [38], VCAM1^+^ [39], and SDC4^+^ [40]. Furthermore, a range of fluorescence-based Pax3-, Myf5-, and Pax7-driven reporter mouse models have permitted the isolation of purified populations of MuSCs using FACS [36, 41–43]. However, these traditional methods for isolating MuSCs are under scrutiny due to several limitations. The reporter models still struggle to distinctly identify and isolate MuSC subpopulations based on a single reporter expression level alone. For instance, MuSCs from the trunk and forelimb muscles express Pax3, whereas those from the hindlimb do not [44, 45]. Although the *Myf5* locus is active in quiescent MuSCs [36], for the Myf5 reporter line, its reporter expression is below detection levels in many of the MuCSs [46–49]. Consequently, the Pax7 reporter line remains widely used to isolate MuSCs. However, numerous technical variabilities, such as differences in animal models, spectral properties, subcellular localization, and protein stability, raise concerns over the ability of Pax7 reporter models to differentiate MuSC subpopulations [17, 49].

The current standard for isolating MuSCs is a two-step enzyme digestion procedure involving a lengthy mechanical and enzymatic tissue dissociation to release mononuclear cells associated with myofibers. Recent studies have revealed that significant global transcriptomic and epigenetic changes are associated with the dissociation and purification of MuSCs using FACS [50–52]. These isolation-induced artifacts introduce strong contaminating signals in bulk data, raising questions on the validity of previously published transcriptomics studies and highlighting the importance of single-cell resolved experiments. In addition, surface marker sorting schemes do not address the equivalency of MuSCs in injured muscles or older animals. Following muscle injury, the phenotype of MuSCs changes significantly (Fig. 1). Specifically, the expression of surface markers such as CD34 and CXCR4 decreases, whereas the expression of Sca-1 increases [42]. In older mice, the frequency of PAX7-expressing MuSCs declines while exhibiting a higher expression level of PAX7. Furthermore, with aging, a shift towards a more heterogeneous, less regenerative, and senescent MuSC population is observed [53].

## Technical challenges and biases in studying MuSC states

Early responses of MuSCs to the disruption of their niche include increased expression of AP-1 members and ERK1/2 targets such as Fos and Jun, rapid downregulation of genes encoding Hox proteins, zinc finger proteins, metabolism enzymes, and Notch signaling [50–52, 54]. Capturing the early in vivo responses of MuSCs presents a significant challenge, as the isolation procedures—through mechanical and enzymatic tissue dissociation—induce major transcriptional modifications. These alterations can obscure the true changes induced by injury.

Indeed, across all tissues in the Tabula Muris, a comprehensive multi-organ murine single-cell atlas, a pronounced positive correlation is evident between the duration of dissociation and the activation of stress-response genes [23, 55]. This underscores the importance of prompt MuSC isolation to prevent the initiation of the myogenic program following muscle disaggregation. In this regard, single-nucleus RNA-sequencing (snRNA-seq) is an excellent alternative to single-cell RNA-sequencing (scRNA-seq) as it minimizes spurious expression of genes resulting from isolation as tissue preparation for snRNA-seq is short and only considers nuclei. The short preparation time, combined with the absence of ribosomes, prevents the translation of mRNAs induced during dissociation [56, 57]. Also, snRNA-seq eliminates the need for intact, viable single cells, thereby enabling the analysis of frozen tissues or individual nuclei from myotubes/myofibers. Unlike the Tabula Muris Limb Muscle dataset, which shows enrichment of dissociation-associated transcripts (*Jun*, *Fos*, and *Egr1*) in MuSCs, these were undetectable in MuSCs from snRNA-seq following dissociation [23, 24]. Likewise, transcriptional alteration is not limited to MuSCs, as dissociation affects the transcriptome of most cell types in skeletal muscle, including stromal cells, endothelial cells, and macrophages [23], highlighting the utility of snRNA-seq in studying muscle regeneration.

One of the key differences observed between scRNA- and snRNA-seq is the proportions of detected gene biotypes. Differential expression analysis performed within identical cell types but comparing between scRNA- and snRNA-seq reveals that transcriptional variations are mainly observed in protein-coding, lncRNA, mitochondrial, ribosomal, heat-shock, and surface protein genes, whereas transcripts encoding transcription factors show minimal differences [24]. Only lncRNAs were elevated in snRNA-seq across all cell types, a notable finding given that some of these lncRNAs are discovered to encode pri-miRNAs that regulate gene transcription and chromatin structure during myogenesis [58]. While snRNA-seq offers significant advantages, including minimizing artifacts from cell dissociation and enabling the analysis of myofibers, its application in capturing the temporal dynamics of muscle regeneration has been limited (Table 2). Given the emerging insights into myofiber heterogeneity from recent single-cell studies [59–62], there is a compelling opportunity to leverage snRNA-seq to explore the cellular interplay between myofiber and other cell types during muscle regeneration.

**Table 2:** List of single-nucleus studies on MuSCs using sorted cells or whole muscles

Studies	Omics type	Cell Isolation procedure	Injury site	Injury model (dpi)	Ref
Machado et al., 2021	snRNA-seq	Whole muscle + FACS (Live)	TA	BaCl_2_ (0, 4 h)	[23]
Jing et al., 2023	snRNA-seq	Whole muscle + FACS (Live)	TA + Quad	CTX (0, 10)	[63]
Lin et al., 2023	snRNA-seq	Whole muscle	GA	Denervation (0, 14)	[64]
	snATAC-seq				
Okafor et al., 2023	snATAC-seq	Whole muscle + FACS (Live)	HL	BaCl_2_ (0, 0.25, 0.5, 1, 2, 3, 7)	[27]

*Abbreviations*: *TA* Tibialis anterior; *Quad* Quadricep, *GA* Gastrocnemius, *HL* Hindlimb

The use of FACS to isolate viable or target cells before scRNA-seq introduces additional limitations. Cell sorting based on Calcein positivity decreased the incidence of myogenic cells and anti-inflammatory macrophages while favoring dendritic cells, T cells, and NK cells, which are more metabolically active [19]. Like FACS, applying fluorescence-activated nucleus sorting (FANS) negatively influenced single-nucleus ATAC sequencing (snATAC-seq) results, showing lower transcription start site enrichment, poorer overlap, and correlation with existing bulk ATAC-seq signals [65]. Interestingly, FANS did not alter snRNA-seq results, as all libraries displayed high pairwise correlations with substantially increased quality of nuclei.

## Quiescence heterogeneity based on expression

One of the first studies to employ single-cell transcriptomic analysis on quiescent MuSCs was conducted using FACS-sorted *Pax7*-tdTomato^+^ MuSCs and the Fluidigm C1 system [66]. Although limited to 21 cells, this study provides a transcriptomic overview of MuSC characteristics and heterogeneity, confirming that quiescent MuSCs display enriched levels of *Sdc4* transcripts, along with variability in pathways related to protein ubiquitination, post-translational modifications, and macromolecule metabolism processes. Another study that performed scRNA-seq analysis on human MuSCs from individuals of varying ages focuses on the diverse heterogeneity of quiescent MuSC [67]. This diversity is evident not only in the differential expression of genes encoding PAX7, SPRY1, MYF5, MYOD1, MYOG, various collagens, and cyclins but also in the differential enrichment of NOTCH, IFN-γ, TNF, and TGF-β signaling pathways. Differential gene expression analysis between *SPRY1* ^hi^/*HEY1* ^hi^ and *SPRY1* ^lo^/*HEY1* ^lo^ MuSC clusters identified CAV1 as closely associated with quiescence. CAV1^+^ cells differed in morphology from CAV1^−^ cells and showed slower division rates and lower expression of *Mki67* and MYOD in vitro, indicating a resistance to activation. Furthermore, CAV1^+^ MuSCs demonstrate a four-fold higher engraftment capacity over CAV1^−^ cells, displaying a greater degree of quiescence. CAV1 is predominantly observed at the surface of CD34^+^ quiescent MuSCs and is downregulated upon differentiation in vitro and in vivo [68]. Outside of MuSCs, CAV1 has been suggested to localize at the base of the primary cilium to regulate cilium size [69] and Hedgehog signaling via mediating the ciliary exit of cilium components [70]. Future studies will elucidate whether CAV1 can regulate the quiescence of MuSCs through the primary cilia (see the section “Intermediate State Between Quiescence and Activation”).

In the context of surface markers, CD34 emerges as a canonical MuSC surface marker used to delineate stages of MuSC quiescence, with CD34^hi^ MuSCs being the most stem population with better engraftment and self-renewal capabilities [36, 52, 71]. CD34^hi^ MuSCs are called genuine quiescent as they are set to self-renew following injury, whereas CD34^lo^ MuSCs, referred to as primed quiescent, are prone to activate and commit to the myogenic program precociously [72–74]. Expanding on these earlier insights, applying cytometry by time of flight (CyTOF) to CD9^+^ MuSCs has uncovered previously unrecognized subpopulations, distinguished by the protein co-expression pattern of CD9 and CD104 [75]. The frequency of engraftment decreases in the order of CD9^lo^/CD104^lo^, CD9^hi^/CD104^lo^, and CD9^hi^/CD104^hi^, with the CD9^hi^/CD104^hi^ subpopulation representing committed MYOD^hi^/MYOG^hi^ myoblasts. Later, the same group performed CyTOF on young (2 months) and aged (24 months) muscles to identify distinct subpopulations in CD34^+^ MuSCs [76]. CD47, also known as the “don’t eat me” signal in efferocytosis, is predominantly increased on the cell surface of aged MuSCs due to alternative polyadenylation in CD47 isoforms. CD47^lo^ MuSC subpopulation, whether isolated from young or aged mice, exhibits greater engraftment than the CD47^hi^ counterpart.

## Mechanisms regulating MuSC quiescence

**Fig. 2:**
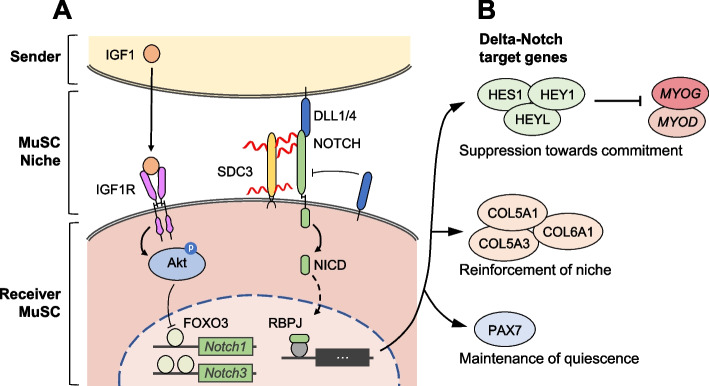
Regulatory mechanisms involved in the maintenance of MuSC quiescence. NOTCH receptors release the Notch intracellular domain (NICD) upon binding to its ligand, DLL1/4. Notch signaling is amplified through the cooperation of SDC with NOTCH receptors and the FOXO signaling pathway (**A**). Transcription factor RBPJ and its moiety NICD regulate the expression of Delta-Notch target genes, which collectively maintain MuSC quiescence (**B**)

A key signaling system involved in the control of MuSC quiescence is the canonical Notch pathway (Fig. 2A). Delta-like canonical Notch ligands 1 and 4 (DLL1/4) are expressed at the fiber membrane and interact with receptors NOTCH1-3 on the MuSC surface [77, 78]. Upon interaction, Notch signaling translocates the Notch intracellular domain (NICD) to the nucleus, activating the transcription factor RBPJ [79]. Notably, the interaction between DLL1/4 and NOTCH receptors requires the cooperation of SDC3 by facilitating the ADAM17 processing of NOTCH receptors. This cooperation is vital for facilitating Notch signal transduction, as demonstrated by the impaired Notch signaling in *Sdc3* knockout MuSCs, leading to premature quiescence exit and lack of self-renewal [80]. These processes positively regulate the direct target genes of Delta-Notch signaling, such as *Pax7*, *Hes1*, *Hey1*, and *Heyl*, maintaining the quiescence of MuSCs (Fig. 2B). Specifically, the deletion of *Hey1* and *Heyl* disrupts the quiescent state, resulting in the re-entry of MuSCs into the cell cycle and the upregulation of myogenic differentiation markers MYOD and MYOG [81]. In parallel, Notch signaling orchestrates the expression of collagen genes, resulting in the autocrine production of collagen V and VI by MuSCs [82, 83]. This production of collagens acts as a feed-forward mechanism, stabilizing the niche and reinforcing the MuSC quiescent state. Specifically, the presence of collagen VI in the culture medium suppresses MuSC differentiation, and the targeted deletion of *Col5a1* in MuSCs leads to a rapid depletion of the MuSC niche [82]. Of note, DLL1, later produced by committed myoblasts, acts as a *cis*-inhibitory ligand to autonomously regulate the balance between self-renewal and differentiation [84, 85].

Previous studies highlight the importance of Notch signaling in MuSCs, yet the distinct roles of individual Notch signaling components are still not fully understood. Current research is beginning to unravel the mechanistic function of the different Notch components [86–88]. Recently, a scRNA-seq study on injured muscle provided insights into the mechanism of the DLL1-NOTCH2 axis in controlling MuSC quiescence [22]. MuSCs at 4 days post-injury have five distinct subpopulations: quiescent *Pax7* ^hi^ state, collagen-enriched state, *Sdc4* ^hi^/*Notch2* ^hi^ state, cyclin-enriched proliferative state, and differentiating *Dll1* ^hi^ state. Pseudotime analysis of the five clusters reveals that commitment to differentiation is marked by a switch from NOTCH2 expression to DLL1 expression, suggesting that the *Notch2* ^hi^ MuSCs may represent a transitional state that plays a crucial role in determining MuSC fate. Notably, in vitro, when NOTCH receptors were blocked individually, only inhibition of NOTCH2 resulted in myotube formation. Additionally, inhibition of both DLL1 and NOTCH2 shifts the cell fate of *Notch2* ^hi^ cells toward a differentiating state at the expense of proliferative and collagen-enriched states. These results suggest that a heterogeneous population of MuSCs mediates self-renewal during regeneration, underscoring the utility of scRNA-seq in elucidating the roles of individual components regulating MuSC quiescence [22].

Stabilization/reactivation of Notch signaling is driven by the transcription factor forkhead box O3 (FOXO3). FOXO3 enhances the expression of *Notch1* and *Notch3* receptors and promotes the quiescent state during MuSC self-renewal (Fig. 2A) [89]. FOXO3 protein level is higher in genuine quiescent MuSCs than primed quiescent MuSCs [73], suggesting the role of FOXO signaling in maintaining stemness in MuSCs. Insulin-like growth factor 1 (IGF1) produced by M2-like macrophages stimulates the PI3K/Akt signaling pathway and downregulates the activity of FOXO, consequently, Notch signaling [90]. The concomitant reduction of growth factors in the microenvironment at the end of the regeneration phase is speculated to induce a synergistic increase in FOXO and Notch signaling that favors MuSC self-renewal.

scRNA-seq analysis of developmental myogenesis from in vivo human pluripotent stem cells reveals that FOXO3- and FOXO-mediated cell cycle pathway is highly enriched in postnatal quiescent MuSCs, suggesting its role in regulating the transition from proliferative prenatal MuSCs to quiescent postnatal MuSCs [91]. A snRNA-seq comparison of young versus old primate muscle discovered that genes related to the NOTCH signaling in MuSC are downregulated with aging [92]. Indeed, *FOXO3* is downregulated in most cell types in aged muscle, along with a reduction in protein levels. FOXO3-deficient cultured human myotubes undergo accelerated aging while constitutively activating the endogenous FOXO3 alleviates its senescence, suggesting a protective role of FOXO3 against progressive muscle degeneration. The same group later identified SESN1 as one of the target genes of FOXO3, which also induces a senescent phenotype after knockout [63]. The exogenous supplementation of recombinant SESN1 rescues the senescent phenotype in cultured FOXO3-deficient human myotube and boosts muscle regeneration in vivo. snRNA-seq of injured muscle treated with recombinant SESN1 confirms the enrichment of pro-regenerative markers in myofiber, FAPs, and interstitial cells.

## Intermediate state between quiescence and activation

**Fig. 3:**
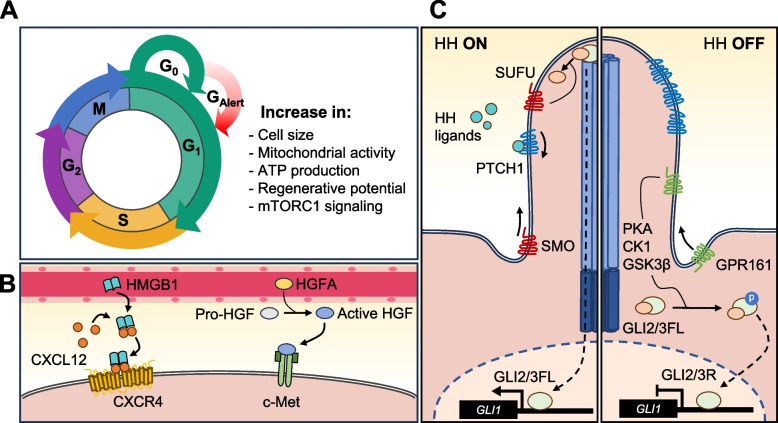
G_Alert_ state. Cells in the G_Alert_ state are characterized by their increase in cell size, mitochondrial activity, ATP production, and most notably, mTORC1 signaling (**A**). Quiescent MuSCs can enter the G_Alert_ state via systemic signals and upregulation of Hedgehog (HH) signaling (**B**, **C**). Circulating fully reduced HMGB1 forms a heterocomplex with CXCL12 homodimer in distal tissues, which binds to the CXCR4 homodimer receptor. Similarly, circulating HGFA proteolytically activates pro-HGF in the ECM or circulatory system, allowing the binding of active HGF to c-Met (**B**). The presence of HH ligands determines post-translational modifications of GLI transcription factors. In the absence of HH, GLI2/3FL is processed into its C-terminally truncated form, repressing HH target genes. In the presence of HH, GLI2/3FL retains its full-length form, activating HH target genes (**C**)

Reflecting their inherent plasticity, MuSCs exhibit a continuum of stages along the quiescence-to-activation trajectory. Quiescent MuSCs can exist in an “alerted” state, termed G_Alert_, representing an intermediate cellular phase between G_0_/quiescent and G_1_/activated. G_Alert_ MuSCs are slightly bigger and are primed for division with increased mitochondrial activity, regenerative potential, and mTORC1 signaling (Fig. 3A) [93]. Tissue damage triggers the hepatocyte growth factor activator (HGFA), leading to the proteolytic cleavage and subsequent activation of HGF [94]. This process stimulates the PI3K/Akt/mTOR pathway through HGF-c-Met signaling transduction to promote the G_Alert_ state in MuSCs and FAPs. Also, high mobility group box 1 (HMGB1) is released upon injury, which forms an HMGB1-CXCL12 complex to shift CXCR4^+^ MuSCs to the G_Alert_ state [95]. HGFA and HMGB1 are circulating factors that increase in concentration following muscle injury (Fig. 3B). Thus, these two factors can partially explain the phenomenon of distal site priming, where an initial injury accelerates the healing of local and distal tissues in response to subsequent trauma [96, 97]. Additionally, it accounts for the cell state of quiescent MuSCs in muscles contralateral to the injury, exhibiting a distinct cycling property compared to naive quiescent and injury-experienced activated MuSCs [93].

The majority of quiescent MuSCs have a single primary cilium, a non-motile microtubule-based structure, which rapidly disassembles upon activation and reassembles preferentially during self-renewal [98]. The primary cilium serves as a cellular antenna for multiple signaling pathways, most notably the Hedgehog (HH) signaling pathway (Fig. 3C). The sequestration of HH pathway components into the cilium regulates the downstream processing of GLI effector proteins [99]. In quiescent MuSCs and differentiating myoblasts, GLI3 is mainly processed as a repressor, while GLI3FL accumulation is observed in proliferative myoblasts [100]. Genetic ablation of *Gli3* in MuSCs induces increased expression of HH target genes (*Gli1* and *Ptch1*), cell size, mitochondrial activity, and mTORC1 signaling, inducing G_Alert_ transition to elevate proliferation at the expense of early differentiation [100].

scRNA-seq reveals that MuSC is one of the cell types that express *Gli1* in homeostatic muscle [101]. GLI1^+^ MuSCs are characterized by their elevated mTORC1 signaling, mitochondrial activity, cell size, and proliferative capacity over GLI1^−^ MuSCs, confirming that they are G_Alert_. Before injury, GLI1^+^ MuSCs make up about 13% of the total MuSC population, but after injury, this proportion doubles to 24% in the contralateral leg. Over 50% of these newly generated GLI1^+^ MuSCs from the contralateral leg are derived from GLI1^+^ MuSCs, suggesting that GLI1^+^ MuSCs significantly contribute to the formation of G_Alert_ cells after injury. Furthermore, scRNA-seq of *Talpid3* knockout and wild-type MuSCs shows that primary cilia are critical in upregulating Wnt and Hedgehog signaling during myogenesis [102]. Interestingly, rescuing Hedgehog signaling in *Talpid3* knockout MuSCs did not restore muscle repair, while rescuing Wnt signaling could. As the G_Alert_ state represents a relatively new concept in stem cell biology, further studies are required to understand how the endogenous HH signaling of primary cilia controls MuSC fate.

## Intercellular communication regulating MuSCs

**Fig. 4:**
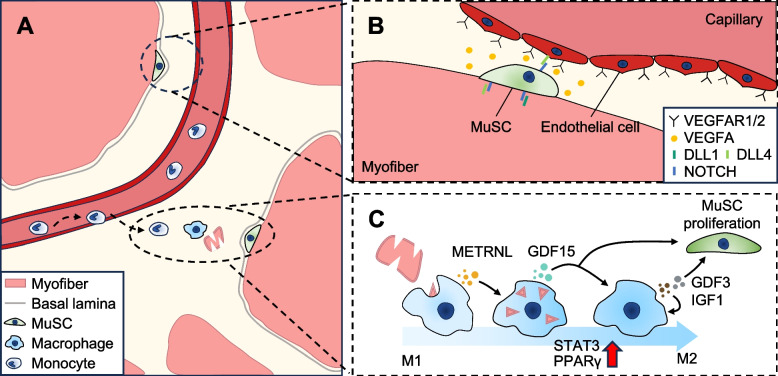
Cell-to-cell communication in injured muscle. **A** Schema depicting a cross-section of a regenerating fascicle. Structural cues and signaling molecules in the niche dictate the cell fate of MuSCs. The immediate microenvironment surrounding the MuSCs is determined by the spatiotemporal presence of other cell types and their secreted cytokines, growth factors, membrane-bound ligands, and ECM constituents. **B** The vascular niche promotes MuSC return to quiescence. Activated MuSCs transiently express VEGFA, which recruits endothelial cells and establishes a vascular niche in their vicinity. Following injury, endothelial cells highly express DLL4, which activates Notch signaling in MuSCs, concomitantly returning to quiescence. The basal lamina, which remains physically intact after muscle injury, provides a spatial outline for the formation of de novo myofibers and capillaries. These nascent myofibers and capillaries give rise to a new layer of basal lamina [105]. The exact nature of the old and new basal lamina during the intercellular communication between MuSCs and endothelial cells—whether the old basal lamina contains “fractures” as suggested by Verma et al. (2018) or the basal lamina itself is selectively permeable to small signaling molecules as seen in the kidney glomerulus [106]—is further to be explored. **C** Phenotype switching of macrophages modulates MuSC proliferation. Following neutrophils, the Ly6C^+^ monocytes infiltrate, engage in efferocytosis to clear necrotic cell debris, and differentiate into Ly6C^−^ macrophages. At 1 dpi, Ly6C^+^ pro-inflammatory macrophages produce METRNL, serving as an autocrine signal that skews these macrophages towards an M2 phenotype. METRNL enhances IGF1 secretion via a STAT3-dependent pathway, aiding MuSC proliferation and sustaining M2-like macrophage activation. By 4 dpi, Ly6C^−^ anti-inflammatory macrophages, functioning in a PPARγ-dependent manner, secrete growth factors GDF15 and GDF3. These factors act paracrinally on MuSCs to promote their proliferation. Notably, GDF15 also has an autocrine role, driving the phenotypic switch of macrophages

Intercellular communication signals, acting through secreted ligands binding to receptors on MuSCs, govern a multitude of cell-fate regulation mechanisms critical for muscle homeostasis and regeneration (Fig. 4A) [103, 104]. Given this scenario, single-cell transcriptomics can be leveraged for data-driven analysis, facilitating the inference of novel ligand-receptor pairs through co-expression patterns. It should be noted that interactome analysis with single-cell transcriptomics does not consider the spatial proximity between cell types, whether proteins are expressed or whether putative interaction pairs are documented specifically within muscle regeneration.

The first study to utilize single-cell transcriptomics on whole muscle tissue to analyze heterotypic cell-to-cell interaction revealed the roles of syndecans in MuSC proliferation [19]. Performing in vitro recombinant treatment tests on cultured MuSCs with putative ligand-receptor pairs reveals that SDC1 and SDC4 are necessary for FGF2/Fn-induced MuSC proliferation; however, only SDC2 is essential for TGF-β1-induced and RSPO3-suppressed MuSC proliferation. These findings further support the non-redundant and essential roles of syndecans during muscle development and regeneration [107, 108]. Large-scale integration of 111 skeletal muscle single-cell and single-nucleus transcriptomics samples, combined with a spatial transcriptomics dataset, provide a comprehensive intercellular communication analysis across all cell populations [24]. FAPs and MuSCs show significantly higher intercellular communication among all cell populations, highlighting the role of FAPs in modulating MuSC quiescence through ECM remodeling.

Of note, spatial transcriptomics preserves the spatial context of cells, providing insights into the local tissue environments and features at a more detailed level. Through spatial neighborhood analysis, it reveals which cell types are mutually attracted or repelled and uncovers how co-localization with different cell populations can alter cellular behavior. While spatial transcriptomics offers valuable insights by preserving cellular spatial context, it is important to recognize its limitations. Most notably, the resolution of spatial transcriptomics techniques that depend on next-generation sequencing is limited by the size of the capture area associated with each unique positional barcode [109]. Therefore, a corresponding scRNA-seq dataset is often utilized as a reference to deduce the types of cells and proportions contributing to the mixture of mRNAs in each capture spot. In addition, the reliability of cell abundance estimations in spatial transcriptomics heavily depends on several factors, including the quality of the reference scRNA-seq dataset, the selection of data preprocessing methods, and the computational algorithm employed [110, 111], highlighting the need for meticulous planning and execution in utilizing spatial transcriptomics for comprehensive and accurate tissue analysis.

Multiple studies have employed ligand-receptor interaction analysis on the single-cell transcriptomic data obtained from whole muscle tissue [21, 26, 28, 112, 113]. However, these interactome results are purely descriptive and lack the functional validation to connect the inferred intercellular communication to the in vivo regenerative milieu. Given the limitations of solely relying on in silico analysis, scRNA-seq can be used as a supplementary tool to validate the expression of ligand-receptor pairs in certain cell types, such as confirming the exclusive expression of the *Dll4* Notch ligand of endothelial cells, which is crucial for maintaining the quiescence of VEGF-expressing MuSCs (Fig. 4B) [114]. Similarly, scRNA-seq has been used to confirm the predominant expression of *Metrnl* in macrophages (Fig. 4C) [115]. METRNL plays a key role in the phenotype switching of M1-like macrophages into M2-like macrophages, which induces the secretion of IGF1 and GDF3 to promote MuSC proliferation [4, 5, 116]. A recent discovery identified growth differentiation factor 15 (GDF15) as another key player in the phenotype switching of macrophages through the PPARγ-RXR-GDF15 axis [117]. scRNA-seq of M2-like macrophages (Ly6C^−^ macrophages at 4 days post-injury) reveals that *Gdf15* ^+^ macrophage cluster co-expresses paracrine growth factors *Igf1* and *Gdf3*. Additionally, GDF15 serves as a paracrine signal to MuSCs, evidenced by the reduced *Pax7* expression in MuSCs from *Gdf15* knockout mice and enhanced proliferation of cultured myoblasts treated with recombinant GDF15. Although, unlike GDF15, METRNL does not directly engage in the paracrine signaling of MuSCs, these findings further emphasize the complex interplay between macrophages and MuSCs during muscle regeneration, mediated by a network of signaling molecules and growth factors.

## Epigenetic regulation of MuSCs during regeneration

Quiescent MuSCs are characterized by highly dense and compacted heterochromatin [30]. However, instead of constitutive heterochromatin, quiescent MuSCs form facultative heterochromatin, meaning that MuSCs can dynamically convert the chromatin state to euchromatin and reactivate the expression of silenced genes [118]. In MuSCs, polycomb repressive complex 2 (PRC2), composed of core subunits EZH1/EZH2, SUZ12, and EED, initiates deposition and maintenance of H3K27me3 marks, whereas histone demethylases UTX, UTY, and JMJD are responsible for removing the histone modifications [119]. EZH2-PRC2 is expressed in proliferative MuSCs but not in quiescent MuSCs and is known to safeguard their transcriptional identity and proliferation. EZH1-PRC2, instead, is preferentially expressed in quiescent MuSCs and maintains its quiescence. UTX removes H3K27me3 marks upon MuSC activation [120], and JMJD2A is recruited to the *MYOG* promoter during differentiation, where it catalyzes the removal of H3K9me3 marks [121].

Single-cell transcriptomics confirms the distinct expression patterns of EZH1 and EZH2 in wild-type MuSCs [122]. *Ezh1* is most abundant in quiescent MuSCs, diminishing post-injury, whereas *Ezh2*, initially low in the quiescent state, increases in activated MuSCs. *Ezh1* knockout MuSCs exhibit precocious activation, marked by their reduced expression of Notch signaling elements, such as *Pax7*, *Hey1*, and *Rbpj*. This phenomenon is attributed to the reduced cilium-associated and Sonic HH signaling genes in *Ezh1* knockout MuSCs, leading to cilium disassembly in unperturbed *Ezh1* knockout MuSCs. Furthermore, scRNA-seq sequencing on MuSC-specific knockouts of *Utx* or *Jmjd3* demonstrates the non-redundant roles of the two histone demethylases [123]. JMJD3 deficiency led to an abundance of early activation gene signatures, contrasting with UTX deficiency, which was associated with proliferative markers. JMJD3-deficient MuSCs isolated after injury showed reduced cell cycle entry, underscoring the role of JMJD3 in transitioning MuSCs to a proliferative state, a phenomenon not observed in UTX-deficient cells. Notably, JMJD3-deficient MuSCs isolated from pre-injury conditions did not exhibit impairment in re-entering the cell cycle, whereas cells from the contralateral leg demonstrated a compromised ability to re-enter the cell cycle. Indeed, one of the target genes of JMJD3 is HAS2, an enzyme responsible for synthesizing hyaluronic acid, which is incorporated into the ECM to render MuSC competent to receive pro-regenerative signals from the microenvironment. In alignment with these findings, treating JMJD3-deficient MuSCs with hyaluronic acid was sufficient for JMJD3-deficient MuSCs to enter the cell cycle, while treatment with exogenous IFN-γ or IL6 was sufficient to impair the cell cycle re-entry. In conclusion, the utilization of single-cell transcriptomics has illuminated the nuanced roles of epigenetic regulators, bridging epigenetic regulation with the maintenance of quiescence via the primary cilium and circulatory factors, offering a comprehensive view of muscle regeneration.

## Mentions

A seminal study that developed Monocle, the first pseudotime inference algorithm, utilized scRNA-seq data from cultured human myoblasts obtained using the C1 Fluidigm system [124]. This study laid the foundation for numerous subsequent studies to explore MuSC dynamics during myogenesis and regeneration through trajectory inference methods [18, 22, 26–28, 64, 125]. While the overall pseudotime trajectories confirm the generally accepted biological model of myogenesis and provide a continuum of hierarchical myogenic populations, it is important to note that these trajectory analyses are primarily descriptive and thus require further functional validation to fully elucidate their biological significance in the context of in vivo muscle regeneration. Also, it is crucial to recognize the computational shortcomings of these methods in fully decoding the stem cell fate. The cyclic and multi-branching properties of MuSCs, where they can transition back from activated to quiescent states through the self-renewal symmetric and asymmetric division [126], challenge the notion of a linear pseudo-time trajectory from quiescent to activated to proliferative state (Fig. 1). Apart from not capturing the dynamic transition of MuSCs, trajectory methods fall short in integrating complex, reversible epigenetic regulation, and varying environmental cues that MuSCs encounter. These limitations underscore the need for a more sophisticated computational method that can fully integrate the multi-omics, nonlinear, and spatiotemporal-dependent aspects of stem cell state transition [127–131]. Moreover, recent advances in single-cell technologies have enabled the parallel analysis of multiple molecular modalities from the same single cell [132]. Multiple attempts have been made to integrate joint single-cell modality datasets in homeostatic muscle and during myogenesis [65, 133]. As of yet, the application of joint single-cell multi-omics analysis in regenerating muscle remains unexplored.

Single-cell technology has been further utilized to uncover insights into lesser-known aspects of MuSC biology, as well. These include investigating the impact of RNA polymerase II promoter-proximal pausing on MuSC self-renewal [134], the role of fatty acid beta-oxidation in MuSC fate determination [135], and studying the cellular mechanisms in pathological conditions such as in muscular dystrophy [29, 60, 112, 113, 125, 136–139], denervation [64], lipid infiltration [25], and limb ischemia [28]. Each topic adds depth to our understanding of MuSCs and their diverse roles in muscle regeneration and disease. Lastly, while our focus is primarily on MuSCs, numerous other cell types participating in muscle regeneration have been investigated via single-cell technologies (see Additional file 1). The roles of resident macrophages [112, 140, 141] and RORγ^+^ Treg cells [142], as well as the phenotype switching [115, 117, 143] and cellular heterogeneity of macrophages [112, 144], stromal cells [20, 136, 145–147], myofiber, spindle fiber, MTJ, and NMJ compartments [59–62], have been explored using single-cell technologies.

Single-cell technology may also help researchers to elucidate the versatility of macrophage phenotypes and the regulatory mechanisms involved in their phenotypic switch. Despite the critical roles of macrophages in regulating the various processes during regeneration, our comprehension of how macrophages change their phenotype through endogenous microenvironmental cues, metabolic reprogramming, and autocrine signaling remains limited [148]. Furthermore, macrophages were categorized into M1 and M2 subsets based on studies related to in vitro and in vivo studies of parasitic infection [149, 150]. However, mounting evidence suggests that in in vivo conditions, M1 and M2 stimuli often coexist, leading to macrophages exhibiting a mixed phenotype, and macrophages may not undergo clonal expansion to sustain their specific phenotype [151–153]. Likewise, monocyte-derived macrophages in the regenerating muscle do not differentially express the canonical M1 and M2 markers [154–156], and macrophage subsets display distinct heterogeneous transcriptional profiles [117, 140] and proliferative capabilities [155, 157], further complicating the process of conducting an unbiased and simultaneous evaluation of all cell populations involved in skeletal muscle regeneration.

## Concluding remarks

Understanding the intricacies of MuSCs is crucial for comprehending muscle regeneration and enhancing treatments for muscle-related diseases. Applying single-cell technologies in homeostatic, injured, and pathological muscle microenvironments has uncovered significant insights into the cellular diversity, fate determination, and intercellular communications of MuSCs. To date, in silico findings from single-cell studies have been mainly descriptive. Moving forward, we expect the seamless integration of multi-omics and spatiotemporal information to further unravel the complex regulatory mechanisms involved in the regenerating muscle.

## Supplementary Information

Additional file 1. Application of single-cell technology in skeletal muscle. #: Refer to the original study for details. Abbreviations: TA, Tibialis anterior; HL, Hindlimb; GA, Gastrocnemius; Quad, Quadricep; VL, Vastus lateralis; NTX, Notexin; CTX, Cardiotoxin; IR, Ischemia–reperfusion; CTLI, Chronic limb-threatening ischemia.

## Data Availability

N/A.

## Abbreviations

MuSC: Muscle satellite cell
scRNA-seq: Single-cell RNA sequencing
CyTOF: Cytometry by the time of flight
ECM: Extracellular matrix
FAP: Fibro-adipogenic progenitor
MTJ: Myotendinous junction
NMJ: Neuromuscular junction
SDC3/4: Syndecan 3/4
CALCR: Calcitonin receptor
CAV1: Caveolin 1
PAX7: Paired box 7
CXCR4: C–X–C motif chemokine receptor 4
MRF: Myogenic regulatory factor
MYF5: Myogenic factor 5
MYOD: Myoblast determination protein 1
MYOG: Myogenin
MRF4: Myogenic regulatory factor 4
PAX3: Paired box 3
MYH3: Myosin heavy chain 3
DLL1/4: Delta-like canonical Notch ligand 1/4
NICD: Notch intracellular domain
dpi: Days post-injury
FOXO3: Forkhead box O3
IGF1: Insulin-like growth factor 1
snRNA-seq: Single-nucleus RNA sequencing
FACS: Fluorescence-activated cell sorting
FANS: Fluorescence-activated nucleus sorting
snATAC-seq: Single-nucleus ATAC sequencing
HGFA: Hepatocyte growth factor activator
HMGB1: High mobility group box 1
HH: Hedgehog
PRC2: Polycomb repressive complex 2

## References

[1] Laumonier T, Menetrey J. Muscle injuries and strategies for improving their repair. J Exp O. 2016;3:15. 10.1186/s40634-016-0051-7 PMC4958098 27447481

[2] Warren GL, Summan M, Gao X, Chapman R, Hulderman T, Simeonova PP. Mechanisms of skeletal muscle injury and repair revealed by gene expression studies in mouse models. J Physiol. 2007;582:825–41. 17478534 10.1113/jphysiol.2007.132373 PMC2075314

[3] Chazaud B, Sonnet C, Lafuste P, Bassez G, Rimaniol A-C, Poron F, et al. Satellite cells attract monocytes and use macrophages as a support to escape apoptosis and enhance muscle growth. J Cell Biol. 2003;163:1133–43. 14662751 10.1083/jcb.200212046 PMC2173611

[4] Tonkin J, Temmerman L, Sampson RD, Gallego-Colon E, Barberi L, Bilbao D, et al. Monocyte/macrophage-derived IGF-1 orchestrates murine skeletal muscle regeneration and modulates autocrine polarization. Mol Ther. 2015;23:1189–200. 25896247 10.1038/mt.2015.66 PMC4817788

[5] Varga T, Mounier R, Patsalos A, Gogolák P, Peloquin M, Horvath A, et al. Macrophage PPARγ, a lipid activated transcription factor controls the growth factor GDF3 and skeletal muscle regeneration. Immunity. 2016;45:1038–51. 27836432 10.1016/j.immuni.2016.10.016 PMC5142832

[6] Morgan JE, Partridge TA. Muscle satellite cells. Int J Biochem Cell Biol. 2003;35:1151–6. 12757751 10.1016/s1357-2725(03)00042-6

[7] Zammit PS, Golding JP, Nagata Y, Hudon V, Partridge TA, Beauchamp JR. Muscle satellite cells adopt divergent fates. J Cell Biol. 2004;166:347–57. 15277541 10.1083/jcb.200312007 PMC2172269

[8] Mann CJ, Perdiguero E, Kharraz Y, Aguilar S, Pessina P, Serrano AL, et al. Aberrant repair and fibrosis development in skeletal muscle. Skeletal Muscle. 2011;1:21. 21798099 10.1186/2044-5040-1-21 PMC3156644

[9] Lemos DR, Babaeijandaghi F, Low M, Chang C-K, Lee ST, Fiore D, et al. Nilotinib reduces muscle fibrosis in chronic muscle injury by promoting TNF-mediated apoptosis of fibro/adipogenic progenitors. Nat Med. 2015;21:786–94. 26053624 10.1038/nm.3869

[10] Kääriäinen M, Järvinen T, Järvinen M, Rantanen J, Kalimo H. Relation between myofibers and connective tissue during muscle injury repair. Scand Med Sci Sports. 2000;10:332–7. 10.1034/j.1600-0838.2000.010006332.x 11085560

[11] Rantanen J, Ranne J, Hurme T, Kalimo H. Denervated segments of injured skeletal muscle fibers are reinnervated by newly formed neuromuscular junctions. J Neuropathol Exp Neurol. 1995;54:188–94. 7876887 10.1097/00005072-199503000-00005

[12] Fukada S, Uezumi A, Ikemoto M, Masuda S, Segawa M, Tanimura N, et al. Molecular signature of quiescent satellite cells in adult skeletal muscle. Stem Cells. 2007;25:2448–59. 17600112 10.1634/stemcells.2007-0019

[13] Gnocchi VF, White RB, Ono Y, Ellis JA, Zammit PS. Further characterisation of the molecular signature of quiescent and activated mouse muscle satellite cells. PLoS ONE. 2009;4:e5205. 19370151 10.1371/journal.pone.0005205 PMC2666265

[14] Lee JY, Qu-Petersen Z, Cao B, Kimura S, Jankowski R, Cummins J, et al. Clonal isolation of muscle-derived cells capable of enhancing muscle regeneration and bone healing. J Cell Biol. 2000;150:1085–100. 10973997 10.1083/jcb.150.5.1085 PMC2175240

[15] Shinin V, Gayraud-Morel B, Gomès D, Tajbakhsh S. Asymmetric division and cosegregation of template DNA strands in adult muscle satellite cells. Nat Cell Biol. 2006;8:677–82. 16799552 10.1038/ncb1425

[16] Kuang S, Rudnicki MA. The emerging biology of satellite cells and their therapeutic potential. Trends Mol Med. 2008;14:82–91. 18218339 10.1016/j.molmed.2007.12.004

[17] Maesner CC, Almada AE, Wagers AJ. Established cell surface markers efficiently isolate highly overlapping populations of skeletal muscle satellite cells by fluorescence-activated cell sorting. Skeletal Muscle. 2016;6:35. 27826411 10.1186/s13395-016-0106-6 PMC5100091

[18] Dell’Orso S, Juan AH, Ko KD, Naz F, Gutierrez-Cruz G, Feng X, et al. Single-cell analysis of adult skeletal muscle stem cells in homeostatic and regenerative conditions. Development. 2019;146(12):dev174177. 10.1242/dev.174177 PMC6602351 30890574

[19] De Micheli AJ, Laurilliard EJ, Heinke CL, Ravichandran H, Fraczek P, Soueid-Baumgarten S, et al. Single-cell analysis of the muscle stem cell hierarchy identifies heterotypic communication signals involved in skeletal muscle regeneration. Cell Rep. 2020;30:3583-3595.e5. 32160558 10.1016/j.celrep.2020.02.067 PMC7091476

[20] Lee DE, McKay LK, Bareja A, Li Y, Khodabukus A, Bursac N, et al. Meteorin-like is an injectable peptide that can enhance regeneration in aged muscle through immune-driven fibro/adipogenic progenitor signaling. Nat Commun. 2022;13:7613. 36494364 10.1038/s41467-022-35390-3 PMC9734561

[21] Oprescu SN, Yue F, Qiu J, Brito LF, Kuang S. Temporal dynamics and heterogeneity of cell populations during skeletal muscle regeneration. iScience. 2020;23:100993. 32248062 10.1016/j.isci.2020.100993 PMC7125354

[22] Yartseva V, Goldstein LD, Rodman J, Kates L, Chen MZ, Chen Y-JJ, et al. Heterogeneity of satellite cells implicates DELTA1/NOTCH2 signaling in self-renewal. Cell Rep. 2020;30:1491-1503.e6. 32023464 10.1016/j.celrep.2019.12.100

[23] Machado L, Geara P, Camps J, Dos Santos M, Teixeira-Clerc F, Van Herck J, et al. Tissue damage induces a conserved stress response that initiates quiescent muscle stem cell activation. Cell Stem Cell. 2021;28:1125-1135.e7. 33609440 10.1016/j.stem.2021.01.017

[24] McKellar DW, Walter LD, Song LT, Mantri M, Wang MFZ, De Vlaminck I, et al. Large-scale integration of single-cell transcriptomic data captures transitional progenitor states in mouse skeletal muscle regeneration. Commun Biol. 2021;4:1280. 34773081 10.1038/s42003-021-02810-x PMC8589952

[25] Xu Z, You W, Chen W, Zhou Y, Nong Q, Valencak TG, et al. Single-cell RNA sequencing and lipidomics reveal cell and lipid dynamics of fat infiltration in skeletal muscle. J cachexia sarcopenia muscle. 2021;12:109–29. 33244879 10.1002/jcsm.12643 PMC7890272

[26] Cutler AA, Pawlikowski B, Wheeler JR, Dalla Betta N, Elston T, O’Rourke R, et al. The regenerating skeletal muscle niche drives satellite cell return to quiescence. iScience. 2022;25:104444. 35733848 10.1016/j.isci.2022.104444 PMC9207300

[27] Okafor AE, Lin X, Situ C, Wei X, Xiang Y, Wei X, et al. Single-cell chromatin accessibility profiling reveals a self-renewing muscle satellite cell state. J Cell Biol. 2023;222: e202211073. 37382627 10.1083/jcb.202211073 PMC10309185

[28] Southerland KW, Xu Y, Peters DT, Lin X, Wei X, Xiang Y, et al. Skeletal muscle regeneration failure in ischemic-damaged limbs is associated with pro-inflammatory macrophages and premature differentiation of satellite cells. Genome Med. 2023;15:95. 37950327 10.1186/s13073-023-01250-y PMC10636829

[29] Young LV, Wakelin G, Cameron AWR, Springer SA, Ross JP, Wolters G, et al. Muscle injury induces a transient senescence-like state that is required for myofiber growth during muscle regeneration. FASEB J. 2022;36: e22587. 36190443 10.1096/fj.202200289RR

[30] Mauro A. Satellite cell of skeletal muscle fibers. J Cell Biol. 1961;9:493–5. 10.1083/jcb.9.2.493 PMC2225012 13768451

[31] Cheung TH, Rando TA. Molecular regulation of stem cell quiescence. Nat Rev Mol Cell Biol. 2013;14:329–40. 23698583 10.1038/nrm3591 PMC3808888

[32] Rocheteau P, Gayraud-Morel B, Siegl-Cachedenier I, Blasco MA, Tajbakhsh S. A subpopulation of adult skeletal muscle stem cells retains all template DNA strands after cell division. Cell. 2012;148:112–25. 22265406 10.1016/j.cell.2011.11.049

[33] Halevy O, Piestun Y, Allouh MZ, Rosser BWC, Rinkevich Y, Reshef R, et al. Pattern of Pax7 expression during myogenesis in the posthatch chicken establishes a model for satellite cell differentiation and renewal. Dev Dyn. 2004;231:489–502. 15390217 10.1002/dvdy.20151

[34] Sambasivan R, Tajbakhsh S. Skeletal muscle stem cell birth and properties. Semin Cell Dev Biol. 2007;18:870–82. 18023213 10.1016/j.semcdb.2007.09.013

[35] Tedesco FS, Dellavalle A, Diaz-Manera J, Messina G, Cossu G. Repairing skeletal muscle: regenerative potential of skeletal muscle stem cells. J Clin Invest. 2010;120:11–9. 20051632 10.1172/JCI40373 PMC2798695

[36] Beauchamp JR, Heslop L, Yu DSW, Tajbakhsh S, Kelly RG, Wernig A, et al. Expression of Cd34 and Myf5 defines the majority of quiescent adult skeletal muscle satellite cells. J Cell Biol. 2000;151:1221–34. 11121437 10.1083/jcb.151.6.1221 PMC2190588

[37] Sacco A, Doyonnas R, Kraft P, Vitorovic S, Blau HM. Self-renewal and expansion of single transplanted muscle stem cells. Nature. 2008;456:502–6. 18806774 10.1038/nature07384 PMC2919355

[38] Sherwood RI, Christensen JL, Conboy IM, Conboy MJ, Rando TA, Weissman IL, et al. Isolation of adult mouse myogenic progenitors. Cell. 2004;119:543–54. 15537543 10.1016/j.cell.2004.10.021

[39] Liu L, Cheung TH, Charville GW, Rando TA. Isolation of skeletal muscle stem cells by fluorescence-activated cell sorting. Nat Protoc. 2015;10:1612–24. 26401916 10.1038/nprot.2015.110 PMC4793971

[40] Tanaka KK, Hall JK, Troy AA, Cornelison DDW, Majka SM, Olwin BB. Syndecan-4-expressing muscle progenitor cells in the SP engraft as satellite cells during muscle regeneration. Cell Stem Cell. 2009;4:217–25. 19265661 10.1016/j.stem.2009.01.016 PMC3142572

[41] Tichy ED, Sidibe DK, Greer CD, Oyster NM, Rompolas P, Rosenthal NA, et al. A robust Pax7EGFP mouse that enables the visualization of dynamic behaviors of muscle stem cells. Skeletal Muscle. 2018;8:27. 30139374 10.1186/s13395-018-0169-7 PMC6107960

[42] Bosnakovski D, Xu Z, Li W, Thet S, Cleaver O, Perlingeiro RCR, et al. Prospective isolation of skeletal muscle stem cells with a Pax7 reporter. Stem Cells. 2008;26:3194–204. 18802040 10.1634/stemcells.2007-1017 PMC4372243

[43] Montarras D, Morgan J, Collins C, Relaix F, Zaffran S, Cumano A, et al. Direct isolation of satellite cells for skeletal muscle regeneration. Science. 2005;309:2064–7. 16141372 10.1126/science.1114758

[44] Kassar-Duchossoy L, Giacone E, Gayraud-Morel B, Jory A, Gomès D, Tajbakhsh S. Pax3/Pax7 mark a novel population of primitive myogenic cells during development. Genes Dev. 2005;19:1426–31. 15964993 10.1101/gad.345505 PMC1151658

[45] Relaix F, Montarras D, Zaffran S, Gayraud-Morel B, Rocancourt D, Tajbakhsh S, et al. Pax3 and Pax7 have distinct and overlapping functions in adult muscle progenitor cells. J Cell Biol. 2006;172:91–102. 16380438 10.1083/jcb.200508044 PMC2063537

[46] Christov C, Chrétien F, Abou-Khalil R, Bassez G, Vallet G, Authier F-J, et al. Muscle satellite cells and endothelial cells: close neighbors and privileged partners. MBoC. 2007;18:1397–409. 17287398 10.1091/mbc.E06-08-0693 PMC1838982

[47] Danoviz ME, Yablonka-Reuveni Z. Skeletal muscle satellite cells: background and methods for isolation and analysis in a primary culture system. In: DiMario JX, editor. Myogenesis. Totowa, NJ: Humana Press; 2012. p. 21–52. 10.1007/978-1-61779-343-1_2 PMC3325159 22130829

[48] Day K, Shefer G, Richardson JB, Enikolopov G, Yablonka-Reuveni Z. Nestin-GFP reporter expression defines the quiescent state of skeletal muscle satellite cells. Dev Biol. 2007;304:246–59. 17239845 10.1016/j.ydbio.2006.12.026 PMC1888564

[49] Ortuste Quiroga HP, Fujimaki S, Ono Y. Pax7 reporter mouse models: a pocket guide for satellite cell research. Eur J Transl Myol. 2023. 10.4081/ejtm.2023.12174. 38112596 10.4081/ejtm.2023.12174 PMC10811643

[50] Van Den Brink SC, Sage F, Vértesy Á, Spanjaard B, Peterson-Maduro J, Baron CS, et al. Single-cell sequencing reveals dissociation-induced gene expression in tissue subpopulations. Nat Methods. 2017;14:935–6. 28960196 10.1038/nmeth.4437

[51] Machado L, Esteves De Lima J, Fabre O, Proux C, Legendre R, Szegedi A, et al. In situ fixation redefines quiescence and early activation of skeletal muscle stem cells. Cell Reports. 2017;21:1982–93. 29141227 10.1016/j.celrep.2017.10.080

[52] Van Velthoven CTJ, De Morree A, Egner IM, Brett JO, Rando TA. Transcriptional profiling of quiescent muscle stem cells in vivo. Cell Rep. 2017;21:1994–2004. 29141228 10.1016/j.celrep.2017.10.037 PMC5711481

[53] Blau HM, Cosgrove BD, Ho ATV. The central role of muscle stem cells in regenerative failure with aging. Nat Med. 2015;21:854–62. 26248268 10.1038/nm.3918 PMC4731230

[54] Pietrosemoli N, Mella S, Yennek S, Baghdadi MB, Sakai H, Sambasivan R, et al. Comparison of multiple transcriptomes exposes unified and divergent features of quiescent and activated skeletal muscle stem cells. Skeletal Muscle. 2017;7:28. 29273087 10.1186/s13395-017-0144-8 PMC5741941

[55] The Tabula Muris Consortium, Overall coordination, Logistical coordination, Organ collection and processing, Library preparation and sequencing, Computational data analysis, et al. Single-cell transcriptomics of 20 mouse organs creates a Tabula Muris. Nature. 2018;562:367–72. 30283141 10.1038/s41586-018-0590-4 PMC6642641

[56] Grindberg RV, Yee-Greenbaum JL, McConnell MJ, Novotny M, O’Shaughnessy AL, Lambert GM, et al. RNA-sequencing from single nuclei. Proc Natl Acad Sci USA. 2013;110:19802–7. 24248345 10.1073/pnas.1319700110 PMC3856806

[57] Lacar B, Linker SB, Jaeger BN, Krishnaswami SR, Barron JJ, Kelder MJE, et al. Nuclear RNA-seq of single neurons reveals molecular signatures of activation. Nat Commun. 2016;7: 11022. 27090946 10.1038/ncomms11022 PMC4838832

[58] Zeng W, Jiang S, Kong X, El-Ali N, Ball AR, Ma CIH, et al. Single-nucleus RNA-seq of differentiating human myoblasts reveals the extent of fate heterogeneity. Nucleic Acids Res. 2016;44(21):e158. 10.1093/nar/gkw739 PMC5137429 27566152

[59] Dos Santos M, Backer S, Saintpierre B, Izac B, Andrieu M, Letourneur F, et al. Single-nucleus RNA-seq and FISH identify coordinated transcriptional activity in mammalian myofibers. Nat Commun. 2020;11:5102. 33037211 10.1038/s41467-020-18789-8 PMC7547110

[60] Kim M, Franke V, Brandt B, Lowenstein ED, Schöwel V, Spuler S, et al. Single-nucleus transcriptomics reveals functional compartmentalization in syncytial skeletal muscle cells. Nat Commun. 2020;11:6375. 33311457 10.1038/s41467-020-20064-9 PMC7732842

[61] Luo L, Ma W, Liang K, Wang Y, Su J, Liu R, et al. Spatial metabolomics reveals skeletal myofiber subtypes. Sci Adv. 2023;9: eadd0455. 36735792 10.1126/sciadv.add0455 PMC10939097

[62] Petrany MJ, Swoboda CO, Sun C, Chetal K, Chen X, Weirauch MT, et al. Single-nucleus RNA-seq identifies transcriptional heterogeneity in multinucleated skeletal myofibers. Nat Commun. 2020;11:6374. 33311464 10.1038/s41467-020-20063-w PMC7733460

[63] Jing Y, Zuo Y, Sun L, Yu Z, Ma S, Hu H, et al. SESN1 is a FOXO3 effector that counteracts human skeletal muscle ageing. Cell Prolif. 2023;56: e13455. 37199024 10.1111/cpr.13455 PMC10212707

[64] Lin H, Peng H, Sun Y, Si M, Wu J, Wang Y, et al. Reprogramming of cis-regulatory networks during skeletal muscle atrophy in male mice. Nat Commun. 2023;14:6581. 37853001 10.1038/s41467-023-42313-3 PMC10584982

[65] Orchard P, Manickam N, Ventresca C, Vadlamudi S, Varshney A, Rai V, et al. Human and rat skeletal muscle single-nuclei multi-omic integrative analyses nominate causal cell types, regulatory elements, and SNPs for complex traits. Genome Res. 2021;31:2258–75. 34815310 10.1101/gr.268482.120 PMC8647829

[66] Cho DS, Doles JD. Single cell transcriptome analysis of muscle satellite cells reveals widespread transcriptional heterogeneity. Gene. 2017;636:54–63. 28893664 10.1016/j.gene.2017.09.014 PMC5659767

[67] Barruet E, Garcia SM, Striedinger K, Wu J, Lee S, Byrnes L, et al. Functionally heterogeneous human satellite cells identified by single cell RNA sequencing. eLife. 2020;9:e51576. 32234209 10.7554/eLife.51576 PMC7164960

[68] Volonte D, Liu Y, Galbiati F. The modulation of caveolin-1 expression controls satellite cell activation during muscle repair. FASEB j. 2005;19:1–36. 15545301 10.1096/fj.04-2215fje

[69] Rangel L, Bernabé-Rubio M, Fernández-Barrera J, Casares-Arias J, Millán J, Alonso MA, et al. Caveolin-1α regulates primary cilium length by controlling RhoA GTPase activity. Sci Rep. 2019;9:1116. 30718762 10.1038/s41598-018-38020-5 PMC6362014

[70] Schou KB, Mogensen JB, Morthorst SK, Nielsen BS, Aleliunaite A, Serra-Marques A, et al. KIF13B establishes a CAV1-enriched microdomain at the ciliary transition zone to promote Sonic hedgehog signalling. Nat Commun. 2017;8: 14177. 28134340 10.1038/ncomms14177 PMC5290278

[71] Sidney LE, Branch MJ, Dunphy SE, Dua HS, Hopkinson A. Concise review: evidence for CD34 as a common marker for diverse progenitors. Stem Cells. 2014;32:1380–9. 24497003 10.1002/stem.1661 PMC4260088

[72] Alfaro LAS, Dick SA, Siegel AL, Anonuevo AS, McNagny KM, Megeney LA, et al. CD34 promotes satellite cell motility and entry into proliferation to facilitate efficient skeletal muscle regeneration. Stem Cells. 2011;29:2030–41. 21997891 10.1002/stem.759 PMC3638793

[73] García-Prat L, Perdiguero E, Alonso-Martín S, Dell’Orso S, Ravichandran S, Brooks SR, et al. FoxO maintains a genuine muscle stem-cell quiescent state until geriatric age. Nat Cell Biol. 2020;22:1307–18. 33106654 10.1038/s41556-020-00593-7

[74] Jankowski RJ, Deasy BM, Cao B, Gates C, Huard J. The role of CD34 expression and cellular fusion in the regeneration capacity of myogenic progenitor cells. J Cell Sci. 2002;115:4361–74. 12376567 10.1242/jcs.00110

[75] Porpiglia E, Samusik N, Ho ATV, Cosgrove BD, Mai T, Davis KL, et al. High-resolution myogenic lineage mapping by single-cell mass cytometry. Nat Cell Biol. 2017;19:558–67. 28414312 10.1038/ncb3507 PMC5728993

[76] Porpiglia E, Mai T, Kraft P, Holbrook CA, de Morree A, Gonzalez VD, et al. Elevated CD47 is a hallmark of dysfunctional aged muscle stem cells that can be targeted to augment regeneration. Cell Stem Cell. 2022;29:1653-1668.e8. 36384141 10.1016/j.stem.2022.10.009 PMC9746883

[77] Conboy IM, Rando TA. The regulation of notch signaling controls satellite cell activation and cell fate determination in postnatal myogenesis. Dev Cell. 2002;3:397–409. 12361602 10.1016/s1534-5807(02)00254-x

[78] Low S, Barnes JL, Zammit PS, Beauchamp JR. Delta-like 4 activates Notch 3 to regulate self-renewal in skeletal muscle stem cells. Stem Cells. 2018;36:458–66. 29230914 10.1002/stem.2757

[79] Wen Y, Bi P, Liu W, Asakura A, Keller C, Kuang S. Constitutive Notch activation upregulates Pax7 and promotes the self-renewal of skeletal muscle satellite cells. Mol Cell Biol. 2012;32:2300–11. 22493066 10.1128/MCB.06753-11 PMC3372272

[80] Pisconti A, Cornelison DDW, Olguín HC, Antwine TL, Olwin BB. Syndecan-3 and Notch cooperate in regulating adult myogenesis. J Cell Biol. 2010;190:427–41. 20696709 10.1083/jcb.201003081 PMC2922652

[81] Fukada S, Yamaguchi M, Kokubo H, Ogawa R, Uezumi A, Yoneda T, et al. Hesr1 and Hesr3 are essential to generate undifferentiated quiescent satellite cells and to maintain satellite cell numbers. Development. 2011;138:4609–19. 21989910 10.1242/dev.067165 PMC3265560

[82] Baghdadi MB, Castel D, Machado L, Fukada S, Birk DE, Relaix F, et al. Reciprocal signalling by Notch-Collagen V-CALCR retains muscle stem cells in their niche. Nature. 2018;557:714–8. 29795344 10.1038/s41586-018-0144-9 PMC5985950

[83] Urciuolo A, Quarta M, Morbidoni V, Gattazzo F, Molon S, Grumati P, et al. Collagen VI regulates satellite cell self-renewal and muscle regeneration. Nat Commun. 2013;4:1964. 23743995 10.1038/ncomms2964 PMC3682802

[84] Zhang H, Shang R, Bi P. Feedback regulation of Notch signaling and myogenesis connected by MyoD–Dll1 axis. PLoS Genet. 2021;17: e1009729. 34370738 10.1371/journal.pgen.1009729 PMC8376015

[85] Zhang Y, Lahmann I, Baum K, Shimojo H, Mourikis P, Wolf J, et al. Oscillations of Delta-like1 regulate the balance between differentiation and maintenance of muscle stem cells. Nat Commun. 2021;12:1318. 33637744 10.1038/s41467-021-21631-4 PMC7910593

[86] Fujimaki S, Seko D, Kitajima Y, Yoshioka K, Tsuchiya Y, Masuda S, et al. Notch1 and Notch2 coordinately regulate stem cell function in the quiescent and activated states of muscle satellite cells. Stem Cells. 2018;36:278–85. 29139178 10.1002/stem.2743

[87] Nandagopal N, Santat LA, LeBon L, Sprinzak D, Bronner ME, Elowitz MB. Dynamic ligand discrimination in the Notch signaling pathway. Cell. 2018;172:869-880.e19. 29398116 10.1016/j.cell.2018.01.002 PMC6414217

[88] Zhou B, Lin W, Long Y, Yang Y, Zhang H, Wu K, et al. Notch signaling pathway: architecture, disease, and therapeutics. Sig Transduct Target Ther. 2022;7:95. 10.1038/s41392-022-00934-y PMC8948217 35332121

[89] Gopinath SD, Webb AE, Brunet A, Rando TA. FOXO3 promotes quiescence in adult muscle stem cells during the process of self-renewal. Stem Cell Reports. 2014;2:414–26. 24749067 10.1016/j.stemcr.2014.02.002 PMC3986584

[90] Lu H, Huang D, Saederup N, Charo IF, Ransohoff RM, Zhou L. Macrophages recruited *via* CCR2 produce insulin-like growth factor-1 to repair acute skeletal muscle injury. FASEB j. 2011;25:358–69. 10.1096/fj.10-171579 PMC3005436 20889618

[91] Xi H, Langerman J, Sabri S, Chien P, Young CS, Younesi S, et al. A human skeletal muscle Atlas identifies the trajectories of stem and progenitor cells across development and from human pluripotent stem cells. Cell Stem Cell. 2020;27:158-176.e10. 32396864 10.1016/j.stem.2020.04.017 PMC7367475

[92] Jing Y, Zuo Y, Yu Y, Sun L, Yu Z, Ma S, et al. Single-nucleus profiling unveils a geroprotective role of the FOXO3 in primate skeletal muscle aging. Protein Cell. 2023;14(7):499–514. 10.1093/procel/pwac061 PMC10305740 36921027

[93] Rodgers JT, King KY, Brett JO, Cromie MJ, Charville GW, Maguire KK, et al. mTORC1 controls the adaptive transition of quiescent stem cells from G0 to GAlert. Nature. 2014;510:393–6. 24870234 10.1038/nature13255 PMC4065227

[94] Rodgers JT, Schroeder MD, Ma C, Rando TA. HGFA is an injury-regulated systemic factor that induces the transition of stem cells into GAlert. Cell Rep. 2017;19:479–86. 28423312 10.1016/j.celrep.2017.03.066 PMC5468096

[95] Lee G, Espirito Santo AI, Zwingenberger S, Cai L, Vogl T, Feldmann M, et al. Fully reduced HMGB1 accelerates the regeneration of multiple tissues by transitioning stem cells to G Alert. Proc Natl Acad Sci USA. 2018;115: 115. 29674451 10.1073/pnas.1802893115 PMC5949009

[96] Davis TA, Longcor JD, Hicok KC, Lennon GG. Prior injury accelerates subsequent wound closure in a mouse model of regeneration. Cell Tissue Res. 2005;320:417–26. 15856306 10.1007/s00441-005-1107-7

[97] Joseph J, Dyson M. The effect of abdominal wounding on the rate of tissue regeneration. Experientia. 1970;26:66–7. 4312705 10.1007/BF01900396

[98] Jaafar Marican NH, Cruz-Migoni SB, Borycki A-G. Asymmetric distribution of primary cilia allocates satellite cells for self-renewal. Stem Cell Rep. 2016;6:798–805. 10.1016/j.stemcr.2016.04.004 PMC4912054 27161363

[99] Mill P, Christensen ST, Pedersen LB. Primary cilia as dynamic and diverse signalling hubs in development and disease. Nat Rev Genet. 2023;24:421–41. 37072495 10.1038/s41576-023-00587-9 PMC7615029

[100] Brun CE, Sincennes M-C, Lin AYT, Hall D, Jarassier W, Feige P, et al. GLI3 regulates muscle stem cell entry into GAlert and self-renewal. Nat Commun. 2022;13:3961. 35803939 10.1038/s41467-022-31695-5 PMC9270324

[101] Peng J, Han L, Liu B, Song J, Wang Y, Wang K, et al. Gli1 marks a sentinel muscle stem cell population for muscle regeneration. Nat Commun. 2023;14:6993. 37914731 10.1038/s41467-023-42837-8 PMC10620419

[102] Martinez-Heredia V, Blackwell D, Sebastian S, Pearson T, Mok GF, Mincarelli L, et al. Absence of the primary cilia formation gene Talpid3 impairs muscle stem cell function. Commun Biol. 2023;6:1121. 37925530 10.1038/s42003-023-05503-9 PMC10625638

[103] Kann AP, Hung M, Krauss RS. Cell–cell contact and signaling in the muscle stem cell niche. Curr Opin Cell Biol. 2021;73:78–83. 34352725 10.1016/j.ceb.2021.06.003 PMC8678169

[104] Yin H, Price F, Rudnicki MA. Satellite cells and the muscle stem cell niche. Physiol Rev. 2013;93:23–67. 23303905 10.1152/physrev.00043.2011 PMC4073943

[105] Vracko R, Benditt EP. Basal lamina: the scaffold for orderly cell replacement. J Cell Biol. 1972;55:406–19. 5076781 10.1083/jcb.55.2.406 PMC2108791

[106] Miner JH. The glomerular basement membrane. Exp Cell Res. 2012;318:973–8. 22410250 10.1016/j.yexcr.2012.02.031 PMC3334451

[107] Cornelison DDW, Wilcox-Adelman SA, Goetinck PF, Rauvala H, Rapraeger AC, Olwin BB. Essential and separable roles for Syndecan-3 and Syndecan-4 in skeletal muscle development and regeneration. Genes Dev. 2004;18:2231–6. 15371336 10.1101/gad.1214204 PMC517515

[108] Rønning SB, Carlson CR, Aronsen JM, Pisconti A, Høst V, Lunde M, et al. Syndecan-4–/– mice have smaller muscle fibers, increased Akt/mTOR/S6K1 and Notch/HES-1 pathways, and alterations in extracellular matrix components. Front Cell Dev Biol. 2020;8: 730. 32850844 10.3389/fcell.2020.00730 PMC7411008

[109] Williams CG, Lee HJ, Asatsuma T, Vento-Tormo R, Haque A. An introduction to spatial transcriptomics for biomedical research. Genome Med. 2022;14:68. 35761361 10.1186/s13073-022-01075-1 PMC9238181

[110] Cobos FA, Panah MJN, Epps J, Long X, Man T-K, Chiu H-S, et al. Effective methods for bulk RNA-seq deconvolution using scnRNA-seq transcriptomes. Genome Biol. 2023;24:177. 37528411 10.1186/s13059-023-03016-6 PMC10394903

[111] Danaher P, Kim Y, Nelson B, Griswold M, Yang Z, Piazza E, et al. Advances in mixed cell deconvolution enable quantification of cell types in spatial transcriptomic data. Nat Commun. 2022;13:385. 35046414 10.1038/s41467-022-28020-5 PMC8770643

[112] Coulis G, Jaime D, Guerrero-Juarez C, Kastenschmidt JM, Farahat PK, Nguyen Q, et al. Single-cell and spatial transcriptomics identify a macrophage population associated with skeletal muscle fibrosis. Sci Adv. 2023;9:eadd9984. 37418531 10.1126/sciadv.add9984 PMC10328414

[113] Saleh KK, Xi H, Switzler C, Skuratovsky E, Romero MA, Chien P, et al. Single cell sequencing maps skeletal muscle cellular diversity as disease severity increases in dystrophic mouse models. iScience. 2022;25:105415. 36388984 10.1016/j.isci.2022.105415 PMC9646951

[114] Verma M, Asakura Y, Murakonda BSR, Pengo T, Latroche C, Chazaud B, et al. Muscle satellite cell cross-talk with a vascular niche maintains quiescence via VEGF and Notch signaling. Cell Stem Cell. 2018;23:530-543.e9. 30290177 10.1016/j.stem.2018.09.007 PMC6178221

[115] Baht GS, Bareja A, Lee DE, Rao RR, Huang R, Huebner JL, et al. Meteorin-like facilitates skeletal muscle repair through a Stat3/IGF-1 mechanism. Nat Metab. 2020;2:278–89. 32694780 10.1038/s42255-020-0184-y PMC7504545

[116] Spadaro O, Camell CD, Bosurgi L, Nguyen KY, Youm Y-H, Rothlin CV, et al. IGF1 shapes macrophage activation in response to immunometabolic challenge. Cell Rep. 2017;19:225–34. 28402847 10.1016/j.celrep.2017.03.046 PMC5513500

[117] Patsalos A, Halasz L, Medina-Serpas MA, Berger WK, Daniel B, Tzerpos P, et al. A growth factor–expressing macrophage subpopulation orchestrates regenerative inflammation via GDF-15. J Exp Med. 2022;219: e20210420. 34846534 10.1084/jem.20210420 PMC8635277

[118] Boonsanay V, Zhang T, Georgieva A, Kostin S, Qi H, Yuan X, et al. Regulation of skeletal muscle stem cell quiescence by Suv4-20h1-dependent facultative heterochromatin formation. Cell Stem Cell. 2016;18:229–42. 26669898 10.1016/j.stem.2015.11.002

[119] Margueron R, Reinberg D. The Polycomb complex PRC2 and its mark in life. Nature. 2011;469:343–9. 21248841 10.1038/nature09784 PMC3760771

[120] Seenundun S, Rampalli S, Liu Q-C, Aziz A, Palii C, Hong S, et al. UTX mediates demethylation of H3K27me3 at muscle-specific genes during myogenesis. EMBO J. 2010;29:1401–11. 20300060 10.1038/emboj.2010.37 PMC2868576

[121] Verrier L, Escaffit F, Chailleux C, Trouche D, Vandromme M. A new isoform of the histone demethylase JMJD2A/KDM4A is required for skeletal muscle differentiation. PLoS Genet. 2011;7: e1001390. 21694756 10.1371/journal.pgen.1001390 PMC3107188

[122] Feng X, Wang AH, Juan AH, Ko KD, Jiang K, Riparini G, et al. Polycomb Ezh1 maintains murine muscle stem cell quiescence through non-canonical regulation of Notch signaling. Dev Cell. 2023;58:1052-1070.e10. 37105173 10.1016/j.devcel.2023.04.005 PMC10330238

[123] Nakka K, Hachmer S, Mokhtari Z, Kovac R, Bandukwala H, Bernard C, et al. JMJD3 activated hyaluronan synthesis drives muscle regeneration in an inflammatory environment. Science. 2022;377:666–9. 35926054 10.1126/science.abm9735

[124] Trapnell C, Cacchiarelli D, Grimsby J, Pokharel P, Li S, Morse M, et al. The dynamics and regulators of cell fate decisions are revealed by pseudotemporal ordering of single cells. Nat Biotechnol. 2014;32:381–6. 24658644 10.1038/nbt.2859 PMC4122333

[125] Chemello F, Wang Z, Li H, McAnally JR, Liu N, Bassel-Duby R, et al. Degenerative and regenerative pathways underlying Duchenne muscular dystrophy revealed by single-nucleus RNA sequencing. Proc Natl Acad Sci USA. 2020;117:29691–701. 33148801 10.1073/pnas.2018391117 PMC7703557

[126] Ancel S, Stuelsatz P, Feige JN. Muscle stem cell quiescence: controlling stemness by staying asleep. Trends Cell Biol. 2021;31:556–68. 33674167 10.1016/j.tcb.2021.02.006

[127] Colomé-Tatché M, Theis FJ. Statistical single cell multi-omics integration. Curr Opin Syst Biol. 2018;7:54–9.

[128] Ludwig CH, Bintu L. Mapping chromatin modifications at the single cell level. Development. 2019;146:dev170217. 31249006 10.1242/dev.170217 PMC6602357

[129] Mayr U, Serra D, Liberali P. Exploring single cells in space and time during tissue development, homeostasis and regeneration. Development. 2019;146:dev176727. 31249009 10.1242/dev.176727

[130] McKenna A, Gagnon JA. Recording development with single cell dynamic lineage tracing. Development. 2019;146:dev169730. 31249005 10.1242/dev.169730 PMC6602349

[131] Chen C, Liao Y, Peng G. Connecting past and present: single-cell lineage tracing. Protein Cell. 2022;13:790–807. 35441356 10.1007/s13238-022-00913-7 PMC9237189

[132] Stuart T, Satija R. Integrative single-cell analysis. Nat Rev Genet. 2019;20:257–72. 30696980 10.1038/s41576-019-0093-7

[133] Dos Santos M, Shah AM, Zhang Y, Bezprozvannaya S, Chen K, Xu L, et al. Opposing gene regulatory programs governing myofiber development and maturation revealed at single nucleus resolution. Nat Commun. 2023;14:4333. 37468485 10.1038/s41467-023-40073-8 PMC10356771

[134] Robinson DCL, Ritso M, Nelson GM, Mokhtari Z, Nakka K, Bandukwala H, et al. Negative elongation factor regulates muscle progenitor expansion for efficient myofiber repair and stem cell pool repopulation. Dev Cell. 2021;56:1014-1029.e7. 33735618 10.1016/j.devcel.2021.02.025 PMC8357161

[135] Yue F, Oprescu SN, Qiu J, Gu L, Zhang L, Chen J, et al. Lipid droplet dynamics regulate adult muscle stem cell fate. Cell Rep. 2022;38: 110267. 35045287 10.1016/j.celrep.2021.110267 PMC9127130

[136] Camps J, Breuls N, Sifrim A, Giarratana N, Corvelyn M, Danti L, et al. Interstitial cell remodeling promotes aberrant adipogenesis in dystrophic muscles. Cell Rep. 2020;31: 107597. 32375047 10.1016/j.celrep.2020.107597

[137] Malecova B, Gatto S, Etxaniz U, Passafaro M, Cortez A, Nicoletti C, et al. Dynamics of cellular states of fibro-adipogenic progenitors during myogenesis and muscular dystrophy. Nat Commun. 2018;9:3670. 30202063 10.1038/s41467-018-06068-6 PMC6131350

[138] Scripture-Adams DD, Chesmore KN, Barthélémy F, Wang RT, Nieves-Rodriguez S, Wang DW, et al. Single nuclei transcriptomics of muscle reveals intra-muscular cell dynamics linked to dystrophin loss and rescue. Commun Biol. 2022;5:989. 36123393 10.1038/s42003-022-03938-0 PMC9485160

[139] Uapinyoying P, Hogarth M, Battacharya S, Mázala DAG, Panchapakesan K, Bönnemann CG, et al. Single-cell transcriptomic analysis of the identity and function of fibro/adipogenic progenitors in healthy and dystrophic muscle. iScience. 2023;26:107479. 37599828 10.1016/j.isci.2023.107479 PMC10432818

[140] Babaeijandaghi F, Cheng R, Kajabadi N, Soliman H, Chang C-K, Smandych J, et al. Metabolic reprogramming of skeletal muscle by resident macrophages points to CSF1R inhibitors as muscular dystrophy therapeutics. Sci Transl Med. 2022;14: eabg7504. 35767650 10.1126/scitranslmed.abg7504

[141] Krasniewski LK, Chakraborty P, Cui C-Y, Mazan-Mamczarz K, Dunn C, Piao Y, et al. Single-cell analysis of skeletal muscle macrophages reveals age-associated functional subpopulations. eLife. 2022;11:e77974. 36259488 10.7554/eLife.77974 PMC9629833

[142] Hanna BS, Wang G, Galván-Peña S, Mann AO, Ramirez RN, Muñoz-Rojas AR, et al. The gut microbiota promotes distal tissue regeneration via RORγ+ regulatory T cell emissaries. Immunity. 2023;56:829-846.e8. 36822206 10.1016/j.immuni.2023.01.033 PMC10101925

[143] Faas M, Ipseiz N, Ackermann J, Culemann S, Grüneboom A, Schröder F, et al. IL-33-induced metabolic reprogramming controls the differentiation of alternatively activated macrophages and the resolution of inflammation. Immunity. 2021;54:2531-2546.e5. 34644537 10.1016/j.immuni.2021.09.010 PMC7617137

[144] Jin RM, Warunek J, Wohlfert EA. Chronic infection stunts macrophage heterogeneity and disrupts immune-mediated myogenesis. JCI Insight. 2018;3: e121549. 30232283 10.1172/jci.insight.121549 PMC6237226

[145] Giordani L, He GJ, Negroni E, Sakai H, Law JYC, Siu MM, et al. High-dimensional single-cell cartography reveals novel skeletal muscle-resident cell populations. Mol Cell. 2019;74:609-621.e6. 30922843 10.1016/j.molcel.2019.02.026

[146] Leinroth AP, Mirando AJ, Rouse D, Kobayahsi Y, Tata PR, Rueckert HE, et al. Identification of distinct non-myogenic skeletal-muscle-resident mesenchymal cell populations. Cell Rep. 2022;39: 110785. 35545045 10.1016/j.celrep.2022.110785 PMC9535675

[147] Scott RW, Arostegui M, Schweitzer R, Rossi FMV, Underhill TM. Hic1 defines quiescent mesenchymal progenitor subpopulations with distinct functions and fates in skeletal muscle regeneration. Cell Stem Cell. 2019;25:797-813.e9. 31809738 10.1016/j.stem.2019.11.004 PMC6941576

[148] Patsalos A, Tzerpos P, Wei X, Nagy L. Myeloid cell diversification during regenerative inflammation: lessons from skeletal muscle. Semin Cell Dev Biol. 2021;119:89–100. 34016524 10.1016/j.semcdb.2021.05.005 PMC8530826

[149] Martinez FO, Gordon S, Locati M, Mantovani A. Transcriptional profiling of the human monocyte-to-macrophage differentiation and polarization: new molecules and patterns of gene expression. J Immunol. 2006;177:7303–11. 17082649 10.4049/jimmunol.177.10.7303

[150] Raes G, Brys L, Dahal BK, Brandt J, Grooten J, Brombacher F, et al. Macrophage galactose-type C-type lectins as novel markers for alternatively activated macrophages elicited by parasitic infections and allergic airway inflammation. J Leukoc Biol. 2004;77:321–7. 15591125 10.1189/jlb.0304212

[151] Martinez FO, Gordon S. The M1 and M2 paradigm of macrophage activation: time for reassessment. F1000Prime Rep. 2014;6:6. 24669294 10.12703/P6-13 PMC3944738

[152] Murray PJ, Allen JE, Biswas SK, Fisher EA, Gilroy DW, Goerdt S, et al. Macrophage activation and polarization: nomenclature and experimental guidelines. Immunity. 2014;41:14–20. 25035950 10.1016/j.immuni.2014.06.008 PMC4123412

[153] Ransohoff RM. A polarizing question: do M1 and M2 microglia exist? Nat Neurosci. 2016;19:987–91. 27459405 10.1038/nn.4338

[154] Novak ML, Weinheimer-Haus EM, Koh TJ. Macrophage activation and skeletal muscle healing following traumatic injury: macrophage activation in muscle trauma. J Pathol. 2014;232:344–55. 24255005 10.1002/path.4301 PMC4019602

[155] Varga T, Mounier R, Horvath A, Cuvellier S, Dumont F, Poliska S, et al. Highly dynamic transcriptional signature of distinct macrophage subsets during sterile inflammation, resolution, and tissue repair. J Immunol. 2016;196:4771–82. 27183604 10.4049/jimmunol.1502490

[156] Wang X, Zhao W, Ransohoff RM, Zhou L. Infiltrating macrophages are broadly activated at the early stage to support acute skeletal muscle injury repair. J Neuroimmunol. 2018;317:55–66. 29325905 10.1016/j.jneuroim.2018.01.004 PMC5835410

[157] Arnold L, Henry A, Poron F, Baba-Amer Y, Van Rooijen N, Plonquet A, et al. Inflammatory monocytes recruited after skeletal muscle injury switch into antiinflammatory macrophages to support myogenesis. J Exp Med. 2007;204:1057–69. 17485518 10.1084/jem.20070075 PMC2118577

